# Muscle Weakness in the Empty and Full Can Tests Cannot Differentiate Rotator Cuff Tear from Cervical Spondylotic Amyotrophy: Pain Provocation is a Useful Finding

**DOI:** 10.2174/1874325001711011081

**Published:** 2017-09-30

**Authors:** Eiichiro Iwata, Hideki Shigematsu, Kazuya Inoue, Takuya Egawa, Yoshihiro Sakamoto, Yasuhito Tanaka

**Affiliations:** 1 Department of Orthopedic Surgery, Nara Medical University, 840 Shijo-cho, Kashiharashi, Nara 634-8522, Japan; 2 Sakamoto Orthopedic Clinic, Nara, Japan

**Keywords:** Biceps, Rotator cuff tear, Cervical spondylotic amyotrophy, Full can test, Empty can test, Pain provocation, Muscle weakness

## Abstract

**Purpose:**

Rotator cuff tears and cervical spondylotic amyotrophy (CSA) are often confused as the main symptom in those with difficulty in shoulder elevation. Empty and full can tests are frequently used for the clinical diagnosis of rotator cuff tears. The aim of the present study was to investigate whether the empty and full can test results can help differentiate rotator cuff tears from CSA.

**Methods:**

Twenty-seven consecutive patients with rotator cuff tears and 25 with CSA were enrolled. We prospectively performed empty and full can tests in patients with rotator cuff tears and CSA. The following signs were considered positive: (a) muscle weakness during the empty can test, (b) muscle weakness during the full can test, (c) pain provocation during the empty can test, and (d) pain provocation during the full can test. We calculated the sensitivity, specificity, positive predictive value (PPV) and negative predictive value (NPV) of rotator cuff tears for each positive finding.

**Results:**

The sensitivity and specificity of each index were as follows (sensitivity, specificity, PPV, NPV): (a) 77.8%, 0%, 45.7%, 0%; (b) 66.7%, 4.0%, 42.9%, 10.0%; (c) 88.9%, 96.0%, 96.0%, 88.9%; and (d) 74.1%, 96.0%, 95.2%, 77.4%. There were significant differences for each index.

**Conclusion:**

Muscle weakness during the empty and full can tests was not useful in differentiating rotator cuff tears from CSA because of low specificity and PPV. However, pain provocation was useful in differentiating these two conditions because of high specificity and PPV.

## 1 INTRODUCTION

The causes of difficulty in shoulder elevation are often difficult to identify, as this complaint might originate from various shoulder and cervical spine disorders. Additionally, the age at occurrence of pathologies of the shoulder and cervical spine is similar, and these specifically occur in the aging population [1, 2] and are often misdiagnosed. Among these, rotator cuff tears and cervical spondylotic amyotrophy (CSA) are often confused, as one of the main symptoms in both is difficulty in shoulder elevation.

CSA is classified as the proximal or distal type [3-5]. The clinical characteristics of the proximal type of CSA include muscle atrophy in the upper extremities, the absence of or insignificant sensory deficits and lower-extremity symptoms, and weakness of the deltoid and biceps muscles, which often causes dropped shoulder syndrome [3-7].

While one of the major symptoms of rotator cuff tears is difficulty in shoulder elevation, rotator cuff tears usually involve the supraspinatus tendon [8, 9]; therefore, the diagnosis of a rotator cuff tear is mainly based on the diagnosis of a torn supraspinatus tendon. The empty and full can tests are frequently used for the clinical diagnosis of rotator cuff tears, which elicit weakness or pain secondary to a torn supraspinatus tendon [10, 11]. Some authors reported the clinical usefulness of the empty and full can tests for determining the presence of a torn supraspinatus tendon [12-14].

Usually, conservative therapy is effective for CSA, but surgery is sometimes needed if conservative therapy is ineffective. The duration of symptoms is one of the risk factors for poor outcomes after surgical treatment [15, 16], so early diagnosis is very important.

If problems with shoulder elevation due to a rotator cuff tear are suspected, the empty and full can tests are performed. We hypothesized that these tests could also be used to differentiate rotator cuff tears from CSA. Thus, the aim of the present study was to investigate whether the empty and full can tests could be used to differentiate rotator cuff tears from CSA.

## 2 MATERIALS AND METHODS

### 2.1 Patients

This study was approved by the institutional review board at our hospital (No.1135). We enrolled consecutive patients with rotator cuff tears who were scheduled to undergo surgery between January 2014 and August 2015 at our hospital (rotator cuff tear group) and those with CSA who had already diagnosed and presented to our clinic (CSA group).

### 2.2 Diagnosis of Rotator Cuff Tears

Rotator cuff tears were diagnosed by an orthopedic surgeon specializing in shoulder surgeries, and the diagnosis was based on the following criteria: (1) Magnetic resonance imaging (MRI) revealed a tear, and (2) the surgeon who operated recognized the tear intraoperatively. We excluded patients with cuff tear arthropathy.

### 2.3 Diagnosis of CSA

Proximal-type CSA was diagnosed by an orthopedic surgeon specializing in spine surgeries and the diagnosis was based on the following criteria: (1) the chief complaint was difficulty in shoulder elevation, no or insignificant sensory deficit, and no lower extremity symptoms; (2) MRI or computed tomography (CT) myelography revealing C5 or C6 nerve or anterior horn compression; and (3) electromyography performed by a neurologist excluded other diseases, such as amyotrophic lateral sclerosis or motor neuron disease.

### 2.4 Evaluation of Empty Can and Full Can Tests

First author (E.I.) performed the empty and full can tests in both rotator cuff tear and CSA patients. The empty can test [12] was performed with the arm elevated to 90º in the scapular plane and in full internal rotation; the full can test [11] was performed with the arm elevated to 90º in the scapular plane and at 45º external rotation. For each test, we evaluated muscle strength and the presence or absence of pain during the maneuver. Muscle strength was determined on the basis of manual muscle testing (MMT) [17]. We determined the presence of muscle weakness if the MMT was less than 3 or if both the MMT was 4 and the strength of the affected side was less than that of the intact contralateral side. Positive signs for each test are as follows: (a) muscle weakness during the empty can test, (b) muscle weakness during the full can test, (c) pain provocation during the empty can test, and (d) pain provocation during the full can test.

These physical findings were assessed in both rotator cuff tear and CSA patients. We assessed these physical examinations in the CSA patients immediately upon diagnosis of CSA and in the rotator cuff tear patients immediately before surgery.

### 2.5 Sensitivity and Specificity of the Empty and Full Can Tests

We calculated the sensitivity, specificity, positive predictive value (PPV) and negative predictive value (NPV) for each physical examination, and statistical analysis was performed using the chi-squared test or Fisher’s exact probability test. In other analyses, differences in quantitative characteristics, such as age, were determined using the Student’s t-test. Differences in qualitative characteristics, such as sex, were analyzed with the chi-squared test. All statistical analyses were carried out with SPSS version 23.0 for Windows (IBM, Armonk, NY, USA). A p value of <0.05 was considered statistically significant.

## 3 RESULTS

### 3.1 Demographics

Twenty-seven patients with rotator cuff tears and 25 with CSA were enrolled in the present study. The rotator cuff tear group comprised 17 men and 10 women; the CSA group comprised 17 men and 8 women. The mean age was 64.1 years and 69.8 years in the rotator cuff tear and CSA groups, respectively. There was a significant difference with respect to age between the groups, but no difference in sex (Table **1**).

### 3.2 Site of Rotator Cuff Tears

The sites of rotator cuff tears are presented in Table **2**. The supraspinatus tendon was involved in all 27 cases.

### 3.3 Sensitivity and Specificity of the Empty and Full Can Tests

The sensitivity, specificity, PPV and NPV of each positive sign are as follows: (a) muscle weakness during the empty can test: sensitivity 77.8%, specificity 0%, PPV 45.7%, NPV 0%; (b) muscle weakness during the full can test: sensitivity 66.7%, specificity 4.0%, PPV 42.9%, NPV 10.0%; (c) pain provocation during the empty can test: sensitivity 88.9%, specificity 96.0%, PPV 96.0%, NPV 88.9%; and (d) pain provocation during the full can test: sensitivity 74.1%, specificity 96.0%, PPV 95.2%, NPV 77.4%. These were statistically significant differences between groups (Table **3**).

## 4 DISCUSSION

In the present study, the empty and full can tests were performed in both rotator cuff tear and CSA patients to investigate whether these two tests could differentiate rotator cuff tears from CSA. Both tests showed low specificity and PPV for muscle weakness; therefore, these findings might show a high positive rate in patients with CSA. Pain provocation showed high specificity and PPV in both tests. In addition, pain in the empty can test showed high sensitivity (88.9%). Similar to previous reports, the full can test was more accurate than was the empty can test for the clinical diagnosis of rotator cuff tears; this was because avoiding of the risk of mechanical impingement can lead to pain provocation [12-14, 18]. However, pain provocation was useful in the differential diagnosis of rotator cuff tear from CSA, and is an important finding in the present study.

Rotator cuff tears usually involve the supraspinatus tendon [8, 9]; therefore, the diagnosis of rotator cuff tear is mainly based on the diagnosis of a torn supraspinatus tendon. In the present study, the supraspinatus tendon was involved in all 27 cases. Jobe *et al.* reported that a test to assess the ability of the affected shoulder to maintain the arm at 90º elevation in the scapular plane and in full internal rotation elicits weakness or pain secondary to a torn supraspinatus tendon (the empty can test) [10]. Subsequently, Kelly *et al.* proposed a new test to assess the function of the supraspinatus tendon to maintain the arm at 90º elevation in the scapular plane and at 45º external rotation and that elicited weakness or pain, and named this the full can test [11]. These two physical examinations are frequently used for the diagnosis of rotator cuff tears. Some authors reported the clinical usefulness of the empty and full can tests for determining the presence of a torn supraspinatus tendon. Itoi *et al.* reported that the full can test may be more beneficial in the clinical setting, considering the pain provocation [13]. Further, another author reported that pain provocation was more frequent during the performance of the empty can test than it was during the full can test, and the full can test more accurately evaluated muscle weakness [12, 14]. Graichen *et al.* reported that a three-dimensional magnetic resonance imaging study demonstrated that internally rotated arm abduction decreases the size of the subacromial space [18]. This avoids the risk of mechanical impingement that leads to pain, and therefore, the full can test is more accurate than the empty can test for the clinical diagnosis of rotator cuff tears.

One of the major symptoms of rotator cuff tear is difficulty in shoulder elevation. This complaint might originate from cervical pathologies. The chief complaint of patients with cervical pathologies, especially CSA, is also difficulty in shoulder elevation [3-7]. In addition, the predilection age for these two conditions is similar, especially in the aging population [1, 2]. Therefore, CSA is often misdiagnosed as rotator cuff tear. Conservative therapy is usually effective in CSA; however, if it is ineffective, surgery is needed [15, 16]. Tauchi *et al.* reported that early surgery was recommended for CSA patients in whom conservative treatment was unsuccessful, based on clinical status before surgery, including symptom duration [15]. Uchida *et al.* reported that a long preoperative period strongly correlated with less improvement in muscle power [16]. Early diagnosis is therefore vital in CSA.

Diagnosis of rotator cuff tears is comprehensively based on physical findings, disease course, and imaging study, such as with magnetic resonance imaging [12, 19, 20]. However, imaging generally causes a delay in diagnosis and entails a high cost, while physical findings are convenient and enable rapid diagnosis. Therefore, we have identified physical findings (*i.e.*, those of empty and full can tests) that would be useful for differentiating between rotator cuff tear and CSA to prevent misdiagnosing CSA as rotator cuff tear, especially on initial diagnosis.

Our study has several limitations. First, we did not evaluate the coexistence of rotator cuff tears and CSA, *i.e.*, patients with rotator cuff tear did not undergo examinations for CSA and patients with CSA did not undergo examinations for rotator cuff tears. Second, we did not evaluate inter- and intra-observer errors. The examiner was only one person who specialized in spine surgery. Third, we did not conduct a power analysis to determine the appropriate sample size before conducting the present study. However, as there was a significant difference for each physical finding, a type 2 error (caused by a small sample size) might not have occurred. Fourth, the examiner was not blinded from the gold standard diagnosis when performing the test. To eliminate these issues, we aim to conduct a similar prospective study in a large cohort.

We suggest that not only evaluation of muscle weakness but also pain provocation should be conducted during the empty and full can tests for the diagnosis of rotator cuff tears. If pain provocation is positive, CSA could be excluded because of the high specificity and PPV of this test. If only muscle weakness is positive, the possibility of CSA should be considered, because of the low specificity and PPV. Therefore, we can prevent the misdiagnosis of CSA as rotator cuff tear and avoid delays in diagnosing CSA.

## CONCLUSION

Muscle weakness in the empty and full can tests could not differentiate rotator cuff tears from CSA because of its low specificity and PPV; however, pain provocation was useful for differentiating these two conditions because of its high specificity and PPV.

## Figures and Tables

**Table 1:** Demographic data of the patients included in the study.

	Rotator cuff tear group	CSA group	P value
Patient number	27	25	-
Sex	male 17, female 10	male 17, female 8	0.703
Age, years (median ± SD)	68 ± 8.3	73 ± 9.0	0.022*

SD, standard deviation; *Statistically significant (P < 0.05).

**Table 2:** Site of rotator cuff tears.

Torn tendons	Patient number
SSP	15
SSP + ISP	5
SSP+SS	3
SSP + ISP + SS	4
Total number	27

SSP, supraspinatus; ISP, infraspinatus; SS, subscapularis

**Table 3:** Sensitivity and specificity for each positive sign.

	Sensitivity	Specificity	PPV	NPV	*P*
Muscle weakness during the ECT	77.8%	0%	45.7%	0%	0.015*
Muscle weakness during the FCT	66.7%	4.0%	42.9%	10.0%	0.008*
Pain provocation during the ECT	88.9%	96.0%	96.0%	88.9%	< 0.001*
Pain provocation during the FCT	74.1%	96.0%	95.2%	77.4%	< 0.001*

ECT, empty can test; FCT, full can test; PPV, positive predictive value;

NPV, negative predictive value; *Statistically significant (*P* < 0.05).
